# Reviewing identity development in young people living with Type 1 Diabetes Mellitus

**DOI:** 10.1002/jad.12412

**Published:** 2024-09-26

**Authors:** Elinda de Klerk, Elmarí Deacon, Esmé van Rensburg

**Affiliations:** ^1^ Community Psychosocial Research (COMPRES), School of Psychosocial Health, Faculty of Health Sciences North‐West University Potchefstroom South Africa; ^2^ Optentia Research Focus Area, Faculty of Humanities North‐West University Potchefstroom South Africa

**Keywords:** chronic condition, critical review, identity development, Type 1 Diabetes Mellitus, young people

## Abstract

**Introduction:**

Type 1 Diabetes Mellitus (T1DM) is a chronic condition increasing among young people. Identity development occurs during an individual's life and can be significantly influenced by a chronic disease such as T1DM. We have critically reviewed the relevant scientific literature to understand young people's identity development with T1DM.

**Methods:**

A critical review design was employed to answer the research question: “What does scientific literature state regarding identity development in young people living with Type 1 Diabetes Mellitus?” Numerous databases were searched to include the most relevant scientific literature to answer the research questions. Boolean operator phrases were ultimately used to search for the literature. The initial screening produced 1319 scientific literature, among which seven articles were analyzed thematically.

**Results:**

Analysis revealed several significant themes: The identity of young people with T1DM develops differently from those without this chronic condition; young people can either incorporate or contain their T1DM in their identity development, highlighting the complex nature of this process; and numerous external factors significantly influence identity development in young people living with a chronic condition such as T1DM.

**Conclusion:**

The study underscores that identity develops differently in young people living with T1DM than in those without chronic conditions. However, the existing studies and literature exploring the experiences of young people living with T1DM have relied on information from caregivers and health professionals. This reliance on secondary sources underscores the urgent need for more research to obtain data from young people living with T1DM as a primary source. Such a shift in research practices is crucial to gaining a more comprehensive and accurate understanding of their experiences.

## Graphical abstract

This critical review explored identity development in young people living with Type 1 Diabetes Mellitus (T1DM) and concluded with three distinct themes. The first finding indicated that identity development in young people remains a complex process. A process that can be different from their peers who do not have the chronic condition. Acknowledging that the process can look different already creates an understanding for the individual going through the process. The findings further revealed that young people often choose between incorporating or containing their diagnosis. When young people incorporate their chronic condition, they accept that it is an aspect of their identity and self‐concept. Ultimately, it does not define who they are. Findings reported that they are likely to have more positive than negative experiences when incorporation happens. In contradiction, when young people choose to contain their chronic condition, it will often lead to negative experiences in their daily lives. The final identified theme highlights the importance of external factors and their possible influence on their decision of containment or incorporation. These external factors include societal perceptions, peer relationships and family dynamics, which are crucial in how they perceive and relate to their chronic condition.

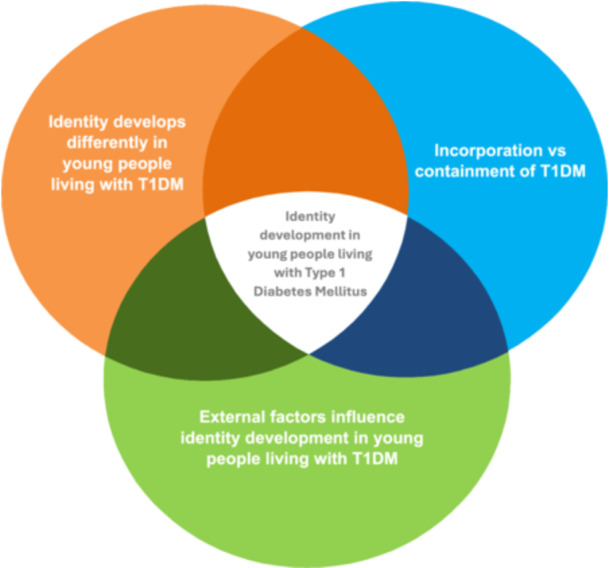

## 1 INTRODUCTION

Diabetes Mellitus is characterized as an intensive, constant and complex chronic condition affecting people of any age and having a significant impact on the individual's life (Chao et al., 2016; Ellis & Jayarajah, 2016). The pathophysiology and management of Type 1 Diabetes Mellitus (T1DM) might seem straightforward. However, DiMeglio et al. (2018) argue that the more we have learned about the disease, the less it seems to be known. Atkinson et al. (2014) report that T1DM is caused by an immune deficiency in which the pancreas struggles to develop β cells, resulting in no insulin production. T1DM is currently treated with a complex regimen of insulin injections, diet and exercise and can significantly affect the lives of young people diagnosed with it and their families (Kakleas et al., 2009). Hagger et al. (2016) describe these effects as diabetes distress, which refers to the negative emotions that can be experienced by young people living with T1DM, and it includes feelings such as frustration, hopelessness, anger, guilt, and fear. These reactions can often be worsened by a lack of understanding or unhelpful interactions with family, friends, and health professionals and by constantly feeling overwhelmed by the demands of managing the condition (Hagger et al., 2016). Apart from the physiological effects of T1DM on young people, their emerging emotional and psychological response to living with the condition also needs attention (Hagger et al., 2016; Kakleas et al., 2009; Wicks et al., 2019).

Identity development is a lifelong process but is specifically prominent during adolescence and emerging adulthood (Vanderhaegen et al., 2024). In addition to identity development, diabetes‐related management is described by Overgaard et al. (2020) as a hardship and burden that young people struggle to balance and uphold. Adolescence and emerging adulthood are crucial life stages that entail several developmental challenges, during which youth often experience self‐management difficulties (Vanderhaegen et al., 2024). Chao et al. (2016), Levesque (2018) and Vanderhaegen et al. (2024) specifically refer to additional stressors which a chronic condition such as T1DM may add to young people's physical and psychological health. These may include influencing a sense of normality, fitting in with peer groups and building new relationships. Jordan et al. (2018) link the interruption of identity development in young people to the stresses induced by T1DM, stating that the chronic condition takes away their identity and the very nature of who they are as individuals.

Although young people living with T1DM have the same developmental issues as healthy young people, Taylor et al. (2008) argue that chronic illness disrupts identity development due to hospitalization, poor health status or changes in appearance. Vanderhaegen et al. (2024) state that youth with a chronic condition such as T1DM often engage in less exploration than their peers living without it. A study by Babler and Strickland (2015) explains that T1DM is the most common chronic illness among young people with multiple physiological and psychological consequences. Over the years, attention has been given to the physiological aspects of living with T1DM; however, the psychological issues have been overlooked (Babler & Strickland, 2015). These psychological issues experienced by young people with T1DM include anxiety, depression, poor self‐esteem, problems with coping and struggles with peer and parental relationships (Babler & Strickland, 2015). Living with T1DM can, therefore, be seen as particularly stressful. It can profoundly impact the person's identity development by eliciting feelings of discontinuity, forcing them to reconsider their sense of self to integrate their condition into their identity (Oris et al., 2016).

Developmental theories describe different periods of life, such as adolescence and adulthood (Erikson, 1968). According to Erikson (1968), as explained by Vanderhaegen et al. (2024, p. 329), identity development can be described as the “conflict between identity synthesis (characterized by adhering to a set of coherent ideals and future goals) and identity confusion (characterized by a lack of meaning and purpose in life).” Furthermore, young adulthood is commonly characterized by adaption to adult roles, gradual separation from parental support, and leaving the parental home (Carlsund & Söderberg, 2019). However, living with a long‐term illness such as T1DM might result in even more challenges for young people who struggle to take full responsibility for their diabetes management (Carlsund & Söderberg, 2019). Young people living with T1DM must juggle and adapt to all the factors while adjusting to the demands of their medical condition (Commissariat et al., 2016). T1DM challenges young people to change and adapt, which may conflict with specific developmental priorities, such as forming an identity (Commissariat et al., 2016; Xing et al., 2015).

Chronic conditions like T1DM affect the physical, psychological, and emotional well‐being of young people, often hindering their ability to manage daily life (Adal et al., 2015). Consequently, these young individuals must adapt to changes in their biology and intellect while managing their treatment (Adal et al., 2015). Therefore, Adal et al. (2015) state that a chronic condition such as T1DM may affect young people's emotional, physical and psychological state, especially in developing an identity (Babler & Strickland, 2015; Crocetti et al., 2013). Furthermore, diabetes self‐management becomes particularly challenging for young people as psychological issues can interfere with the attainment of skills such as coping behaviors (Bernstein et al., 2013). Even so, the great majority of studies on the mental health and psychological aspects of young people living with T1DM have been on a small scale, and there is a strong need for further research to understand the phenomena (Sivertsen et al., 2014). Vanderhaegen et al. (2024) concluded that having TIDM and dealing with an intensive treatment regimen can complicate identity development and processes, making young people feel restricted in their ability to explore and commit to future options in significant life areas. Ultimately, Phase 1 of Carnwell and Daly (2001), which defines the purpose of the review, formed part of the introduction to identify gaps in current scientific knowledge that the critical review addressed. The phases of Carnwell and Daly (2001) are a series of structured stages designed to guide the review process systematically. These phases, which include the identification, synthesis, and evaluation of relevant literature, will serve as a framework to ensure a comprehensive analysis of identity development in young people living with T1DM.

## 2 PURPOSE OF THE PRESENT STUDY

Hussein et al. (2024) explain that the adult brain can understand and make choices about managing diabetes. However, the adolescent brain is processing information more primitively. Going through adolescence and young adulthood with a chronic condition such as T1DM requires self‐management while balancing both cognitive and psychological functioning (Hussein et al., 2024). Hussein et al. (2024) further explain that identity development during adolescence and young adulthood affects various health issues. This includes poor cognitive functioning, hormonal levels affecting disease parameters and lastly, psychological distress (Hussein et al., 2024). Moreover, young people living with T1DM demonstrate a greater incidence of developing depression, anxiety or other forms of psychological distress compared to their healthy peers (Adal et al., 2015; Delamater et al., 2014; Sivertsen et al., 2014). Previous psychological challenges might also hinder identity development in young people living with T1DM, who might, therefore, have difficulty incorporating their diagnosis within their identity (Delamater et al., 2014). This critical review research study aimed to explore the scientific literature regarding identity development in young people with T1DM. The study reported here aimed to analyze the scientific literature critically, present new knowledge and report current national and international findings. Additionally, this study identifies gaps in the scientific literature and recommends further research. We also attempted to add knowledge in psychology and diabetes by answering the following research question: *What does the scientific literature state regarding identity development in young people living with Type 1 Diabetes Mellitus?*

## 3 METHOD

### 3.1 Research design

The purpose of a literature review is to appraise critically and synthesize the current scientific literature relating to the topic being explored (Carnwell & Daly, 2001). The critical review acts as a means of identifying gaps in the knowledge that future studies will seek to address. A critical review was employed for the research study. This critical review assisted the researchers in presenting new findings that will contribute to future research on the topic. As Carnwell and Daly (2001) proposed, guidelines were utilized to conduct this critical review: *Phase 1: Define the purpose of the review*. The preliminary review established a clear purpose for the study and informed the development of the research proposal and data collection process, ensuring the study was well‐grounded and feasible. The literature review included an initial survey of existing research to identify knowledge gaps and define the study's purpose. This approach balanced a thorough understanding of current knowledge with the methodological flexibility required for review research, ensuring a robust and well‐informed foundation. *Phase 2: Defining the scope of the review*. The purpose of the review was defined after identifying current gaps in the literature. The authors incorporated both empirical works and theoretical pieces. Empirical studies provided data‐driven insights and evidence, while theoretical works offered a deeper understanding of relevant discourses, conceptual frameworks, and models. Including both types of literature ensured a well‐rounded examination of the subject. The review focused on published works in peer‐reviewed scholarly journals, ensuring the inclusion of rigorously vetted research.

*Phase 3: Identifying and selecting the sources of relevant information*. Establishing explicit inclusion and exclusion criteria was essential for providing a transparent framework for determining the relevance and significance of the works included in the literature review. These criteria ensured that the review was focused and methodologically sound. The criteria were used to identify an initial pool of 304 potential sources, which was narrowed down to 13 studies through a detailed scrutiny process. The following inclusion criteria enhanced transparency and relevance in the review: (1) Type of Studies: Only empirical studies and theoretical works directly related to the research question were included. (2) Source Type: Only peer‐reviewed literature was considered. (3) Date Range: Studies published within the last 10 years were included to reflect current knowledge and practices.

Exclusion criteria included: (4) Irrelevant Scientific Literature: Studies focusing on constructs not directly related to the research question were excluded to maintain focus. (5) Non‐English Publications: To ensure accessibility and consistency in interpretation, non‐English publications were excluded. Clearly outlining these criteria provided a systematic and justifiable basis for including or excluding studies, ensuring a robust and transparent synthesis of the relevant literature. This approach clarified the decision‐making process and kept the review focused and relevant to the research question.

*Phase 4: Reviewing the literature*. Braun and Clarke's (2006) thematic analysis is a methodical approach to identifying, analyzing, and reporting patterns (themes) within data. It involves six distinct phases: (1) familiarization with the data, where researchers immerse themselves in the data to gain a deep understanding; (2) generating initial codes, which involves systematically coding exciting features across the data set; (3) searching for themes, where codes are collated into potential themes; (4) reviewing themes, involving the refinement of themes to ensure they accurately reflect the data set; (5) defining and naming themes, where each theme is clearly defined and named; and (6) producing the report, which involves the final analysis and writing up of the findings. This structured yet flexible approach allows for a detailed and nuanced understanding of qualitative data, making it a valuable tool in psychological and social research contexts (Braun & Clarke, 2006).

*Phase 5: Writing the review*. After analyses were completed and themes identified, the critical review was written to understand the findings. The process began with an introduction outlining the research objectives and the significance of the thematic analysis. Each identified theme was then presented in detail. The review critically engaged with the themes, discussing their implications, potential limitations, and relevance in existing literature. This reflective and evaluative approach ensured that the review presented and situated themes within broader academic debates, providing a holistic and insightful critique of the research findings.

Lastly, *Phase 6: Applies the literature to the proposed study*. In the final step of a critical review, the theme summaries were integrated into a comprehensive conclusion that logically led to the purpose of a new study and a possible conceptual framework. This involved synthesizing conclusions from all categories into main conclusions, highlighting gaps and shortcomings in previous works, and explaining why these did not address the research question requiring investigation.

### 3.2 Search strategy

Phase 2 (define the scope of the review) and Phase 3 (identify and select the sources of relevant information) of Carnwell and Daly's (2001) guidelines were implemented in the search strategy. Appropriate scientific literature was retrieved from numerous databases, including ScienceDirect, Google Scholar, PsycARTICLES, PsycINFO, EBSCOhost, JSTOR journals, etc. The primary reviewer (first author) independently performed the search, while the second reviewer (second author) monitored the review process and acted as a co‐analyst of extracted data. As there are no formal requirements to present methods of search, synthesis and analysis within a critical review (Grant & Booth, 2009), a simple analytical framework similar to SALSA (Search, Appraisal, Synthesis, and Analysis) was used to explore identity development in young people living with T1DM. The following keywords were used in the search: “identity/identity development/identity formation,” “adolescents/adolescents/young people/youth,” and “Type 1 Diabetes.” Boolean operators were used to help clarify the search. A librarian specialist was consulted to assist in the process.

Studies were included if they were entries from 2014 to 2024, full‐text, peer‐reviewed articles/theses/dissertations, and written in English. The search initially yielded 1319 studies, of which seven were finally included. Scientific literature was further reviewed and excluded if it included children (does not fall within the target population), was outside the time frame and did not speak to identity development. It was further excluded if it included a chronic condition other than T1DM. The studies were excluded if it was irrelevant scientific literature and non‐English studies. The final studies were included to ensure that the research question was answered. See Table 1 for data extraction.

**Table 1:** Summary of extracted data.

	Authors, year and title of article	Study approach, method, and sample	Study results
1.	Commissariat et al. (2016). *Developing a personal and social identity with Type 1 Diabetes during adolescence: A hypothesis generative study*.	*Approach*: Mixed Method. *Measurements*: Audio‐recorded interviews and surveys. *Sample*: 85 participants (ages: 13−20).	Overall, the results showed that T1DM burdened young people's daily lives. In this context, Burden means it hurts their social life, relationships, etc. However, they stated that once they established a routine and managed some of the responsibilities of T1DM, they felt more in control. Young people who incorporated their T1DM and saw the condition as part of their identities were better functioning. Furthermore, those young people who describe successful incorporation of their condition into their identity mentioned the following positive aspects: (1) feelings of mastery and responsibility before their diagnosis, (2) not caring what peers or other people think, (3) being more apprehensive and cautious, (4) hoping to help other people, (5) and feeling overall happier and more confident. Young people who did not incorporate their T1DM into their identities struggled to accept the various aspects of living with T1DM. Young people who only contained their condition and did not incorporate it into their identity struggled with self‐acceptance and maintaining relationships.
2.	Oris et al. (2016). *Illness identity in adolescents and emerging adults with Type 1 Diabetes: Introducing the Illness Identity Questionnaire*	*Approach*: Quantitative. *Measurements*: Condition Identity Questionnaire (CIQ), Center for Epidemiological Studies Depression Scale (CEDS), Satisfaction with Life Scale (SWLS), Self‐Care Inventory (SCI), and Problem Areas in Diabetes Scale (PAID). *Sample*: 575 participants (ages: 14−25).	The study found that diabetes can significantly impact an individual's daily life and hinder other identity‐related obstacles, for example, romantic relationships and educational exploration. They also argue that young people are more prone to struggle with their T1DM if they do not incorporate it within their identity. Young people tend to reject their diagnosis to maintain a “healthy identity.” In other words, if they ignore their diagnosis, they are more likely to struggle with treatment and glycemic control. In contrast with the statement mentioned above, young people who incorporate and accept their diagnosis tend to be happier, with fewer depressive symptoms. Once young people accept their diagnosis of T1DM, they will be better prepared to handle diabetes‐specific related challenges. This will, in return, help them manage their diabetes in the transition to adulthood. Hence, the study supports the importance of incorporating T1DM into young people's identities. Once the individual has accepted and incorporated the diagnosis into their identity, they can deal with other diabetes‐related problems.
3.	Verschueren et al. (2020). *Identity formation in adolescents and emerging adults with Type 1 Diabetes*.	*Approach*: Quantitative. *Measurements*: Dimensions of Identity Development Scale (DIDS); CEDS; SWLS; PAID; Condition Perception Questionnaire (IPQ); The SCI‐Revised (SCI‐R). *Sample*: 431 participants (ages: 14−25).	The authors acknowledge that living with T1DM can challenge young people trying to establish an identity. The study's results suggested that once the individual enters adolescence, there will be a gradual shift in diabetes care responsibilities from the parents to the young people. Young people who successfully experienced achievement and foreclosure in their identity development may experience overall greater control in their lives and mastery over their chronic condition. Individuals who had trouble with their diffusion and moratorium reported having difficulty integrating their T1DM with their identity. Although most individuals can take responsibility for their condition, they often struggle or have difficulty doing so. These difficulties can be linked explicitly to diabetes‐related issues. The study also found that young people with firm commitments to their beliefs and value systems could explore their identity about their chronic condition. Young people in the diffusion and moratorium status struggled most to incorporate their condition into their identity or as a part of themselves.
4.	Commissariat et al. (2019). *Identity and treatment adherence in predominantly ethnic minority teens and young adults with Type 1 Diabetes*.	*Approach*: Mixed‐Method. *Measurements*: Audio‐recorded semi‐structured interviews, self‐reported surveys, and questionnaires. The Texas Social Behavior Inventory Short Form A (TSBI‐A); The Friend Attribution Questionnaire; The Diabetes Social Support Questionnaires (DSSQ‐Friends): The Diabetes Specific Self‐Esteem Scale (DSSE); SCI‐R. *Sample*: 83 participants (ages: 13−21).	Young people who incorporated their condition into their identity were notably more socially competent. Those young people who incorporated their condition had higher self‐esteem, more excellent self‐care, more life satisfaction, and better glycaemic control. Those individuals who did not incorporate their condition into their identity view T1DM as a burden and embarrassment. The study can be divided into two main ideas: young people incorporate their condition into their identity or do not. The study suggested that there can be three significant components of incorporation: (1) acceptance, (2) vocalizing diabetes experience and knowledge and (3) self‐care in the face of stigma. Once the condition became part of their identity, the young people felt more assured in managing their diabetes, experienced greater life satisfaction, and engaged in more excellent self‐care. Lastly, the young people who incorporated their condition had better glycemic control.
5.	Raymaekers et al. (2019). *The social context and condition identity in youth with Type 1 Diabetes: A three‐wave longitudinal study*.	*Approach*: Quantitative. *Measurements*: CIQ, The Inventory of Parent and Peer Attachment (IPPA), The Extreme Peer Orientation Questionnaire, and Child Report of Parent Behavior Inventory. *Sample*: 381 participants (ages: 14−17).	Young people living with T1DM are confronted with various complex tasks, including incorporating their condition into their identity. It is noted that illness identity is how a condition becomes part of one's identity. The study found that overprotective parents may hinder young people's confidence in managing their T1DM. However, caregivers need proper guidance to ensure young people physically and psychologically care for themselves. Lastly, if young people integrate their condition into their identity, it becomes easier for them to engage with peers healthily, and they will likely establish healthier relationships. However, young people may develop their T1DM condition if they become aware that they will not be accepted by their peer groups, leading them to start neglecting their diabetes care routine. This suggests that T1DM can dominate every aspect of young people's lives.
6.	Commissariat et al. (2023). *Assessing incorporation of Type 1 Diabetes into identity: Validation of the Accepting Diabetes and Personal Treatment (ADAPT) survey in teens and young adults*.	*Approach*: Quantitative. *Measurement*: Participants completed the ADAPT survey and validated measures of fear of hypoglycemia, diabetes distress and quality of life. *Sample*: 165 participants (ages: 13−25).	Identity exploration is a hallmark of young people that can conflict with T1DM management. Incorporating T1DM into one's identity has positively affected biomedical and psychosocial outcomes. Enhanced integration of diabetes into one's identity is associated with improved glycemic control, better psychosocial results, and significant benefits related to diabetes management and socio‐demographic factors. This study's findings indicate that a positive self‐identity, when incorporating diabetes, leads to enhanced physical and mental well‐being. Evaluating and strengthening the integration of diabetes into an individual's identity may be crucial for supporting young people to attain improved biomedical and psychosocial outcomes during this challenging developmental phase. Embracing diabetes as an integral part of one's identity and daily routine may decrease distress, as it is perceived as a more regular and manageable aspect of life rather than a burden. Individuals who successfully incorporate diabetes into their daily routine might experience a reduced sense of burden associated with the condition. This, in turn, can lead to a more accepting attitude towards T1DM as merely a component of oneself, eliciting fewer consistently adverse reactions. Consequently, with fewer disruptions, there is a potential for increased involvement in diabetes management, thereby contributing to improved glycemic outcomes.
7.	Ingersgaard et al. (2024). “It's a part of what I am, but not all who I am”: *A qualitative study of identity formation in adolescents and emerging adults with Type 1 Diabetes*.	*Approach*: Qualitative. *Measurements*: Semi‐structured interviews. *Sample*: 15 participants (ages: 15−25).	The formation and development of identity varied among participants as they attributed distinct meanings and significance to diabetes. The themes include the following: (1) internal integration and external separation, (2) normality and deviance, and (3) being in control or being controlled. From this viewpoint, they regarded diabetes as a noteworthy element shaping their sense of identity, influencing how they viewed themselves internally. However, they underscored that diabetes was merely a modest yet meaningful aspect within a broader context. It did not encompass their entire identity. They firmly asserted that they did not define themselves solely by their diabetes. The participants' identity construction within the diabetes framework was closely linked to their perceptions of “normalcy.” Their determination to live lives deemed “normal” was steadfast, and they aimed to harmonize their identity with this standard. Society has imposed specific expectations and assumptions regarding the experience of living with diabetes, and these societal norms often diverge from the personal realities of individuals grappling with the condition. The perceived discrepancy between societal expectations and intimate encounters has led to a sense of stigma and marginalization for those with diabetes. Consequently, they collectively desire to establish a new narrative that recognizes the capacity of individuals with diabetes to lead entirely “normal” lives. The participants emphasized the importance of acknowledging that people with diabetes can actively live, work, and participate in various activities, just like anyone else, without being unduly restricted by their health condition. Despite this aspiration for normalcy, some participants shared their experiences of feeling distinct due to their diabetes. For some, this sense of difference was an enduring aspect. In contrast, others only became aware of it when the unique challenges and demands of diabetes became apparent to those around them. Various situations, especially when dietary restrictions became noticeable during social interactions, triggered feelings of negative distinction. Participants highlighted the difficulty of being told what they could not eat, noting that such restrictions often resulted in feelings of sadness and isolation.

Abbreviations: T1DM, Type 1 Diabetes Mellitus.

### 3.3 Rigour and ethical considerations

For this critical review, it is essential to emphasise that the researchers adhered to the ethical guidelines outlined by the Health Professions Council of South Africa [HPCSA] (Health Professions Act 56 of 1974, SA) during the study. The guidelines followed encompass (1) the reviewer's adherence to the APA guidelines for referencing literature to prevent plagiarism; (2) both the primary and secondary reviewers (first and second author) conducted independent assessments of all scientific literature retrieved, with any uncertainties addressed through consultation with a co‐reviewer (third author) to minimize potential biases; (3) adherence to inclusion and exclusion criteria, which specified the utilization of scientific literature maintaining high ethical standards to prevent ethical misconduct and ensured the reviewed scientific literature received proper ethical clearance. Moreover, (4) ethical clearance was secured from the Health Research Ethics Committee (HREC) of the North‐West University (NWU‐00345‐20‐A1), South Africa.

### 3.4 Data analysis

In Phase 4 of the critical review, according to Carnwell and Daly (2001), thematic analysis was used to generate themes. As explained in the research design section, Braun and Clarke's (2006) guidelines were utilized when conducting thematic analyses.

## 4 FINDINGS AND DISCUSSION

Although the authors searched for theoretical and empirical studies, the final included articles were only empirical. Emphasizing the lack and need for theoretical research on the topic of identity development in young people living with T1DM. After the thematic analysis, the following themes emerged from the data: *Theme 1: Identity develops differently in young people living with Type 1 Diabetes Mellitus. Theme 2: Young people living with Type 1 Diabetes Mellitus can either incorporate or contain their chronic condition in their identity development. Theme 3: External factors influence identity development in young people living with Type 1 Diabetes Mellitus*. Throughout the findings and discussion section of this critical review, Carnwell and Daly's (2001) Phase 5 (writing the review) and Phase 6 (applying the literature to the proposed study) guidelines for conducting a critical review were followed.

Theme 1 Identity Develops Differently in Young People Living with T1DM

All seven articles emphasise the central role of identity development in young people (Commissariat et al., 2016., 2019, 2023; Ingersgaard et al., 2024; Oris et al., 2016; Raymaekers et al., 2019; Verschueren et al., 2020). Furthermore, identity development is prominent in young people, especially when establishing themselves in various roles. The adolescent phase is when the individual attempts to create a meaningful and unique definition of who they are as a person (Commissariat et al., 2019). Although identity development is salient in adolescence and adulthood, it is essential to note that it does not end there (Sugarman, 2004; Topolewska‐Siedzik & Cieciuch, 2019). Identity development continues throughout an individual's lifespan and goes through adulthood into old age (Sugarman, 2004; Topolewska‐Siedzik & Cieciuch, 2019). However, the transition to adulthood for young individuals with diabetes is an interpersonal and spontaneous process that entails redefining supportive relationships with significant others and reconsidering their sense of self (Sparud‐Lundin et al., 2010). Consequently, young people living with T1DM have added layers of complexity to an already transformative time of life, putting them at higher risk of poor self‐care, poor glycaemic control and more severe physiological and psychological complications (Montali et al., 2022).

Young people who carry the burden of T1DM may experience reduced feelings of self‐concept and the ability to form a coherent identity (Raymaekers et al., 2019; Verschueren et al., 2020), leading them to face an additional identity challenge (Vanderhaegen et al., 2024). Themes that emerged from the data indicate that young people who live with T1DM develop an identity status different from those who do not have a chronic condition such as T1DM (Commissariat et al., 2019, 2023; Oris et al., 2016; Raymaekers et al., 2019; Verschueren et al., 2020). While the adolescent is in a transitional period between childhood and adulthood, a chronic condition such as T1DM may have long‐lasting effects on identity development among young people (Raymaekers et al., 2019).

Three of the studies argue that these differences can be observed in the distribution across the various identity statuses (explained by literature as normal identity statuses) throughout their development (Oris et al., 2016; Raymaekers et al., 2019; Verschueren et al., 2020). Young people with T1DM develop an identity differently, affecting their identity statuses and development (Commissariat et al., 2023; Oris et al., 2016; Verschueren et al., 2020). Creating an identity different in this context can be explained by Jones and Foli (2018) and Verschueren et al. (2020) as young people getting used to their chronic condition in their self‐concept and perspective, exploring various roles in their environment along with the difficulty in making accurate decisions for their future.

Verschueren et al. (2020), Commissariat et al. (2019) and Raymaekers et al. (2019), inspired by the work of Erikson (1968) and Marcia (1966), identify the following statuses as being crucial for young people to develop an identity successfully. These statuses include *(1) achievement status*, *(2) foreclosure status*, *(3) moratorium status* and lastly, *(4) diffusion status* (Commissariat et al., 2019; Erikson, 1968; Goethals et al., 2017; Marcia, 1966; Raymaekers et al., 2019). Verschueren et al. (2020) explain that any interruption in identity status may affect young people's overall development, but there is an even more significant effect on their identity development. Ultimately, living with a chronic condition like T1DM can force people to adapt and reshape their identity (Verschueren et al., 2020).

Additionally, to confirm that identity develops differently when living with T1DM, Oris et al. (2016) propose four different identity statuses compared to that of Erikson's (1968) and Marcia's (1966) earlier works: *(1) engulfment* refers to the extent that young people defined themselves in terms of T1DM. When young people define themselves entirely in terms of their condition, it occupies their entire being; *(2) rejection* refers to when young people reject their T1DM, it can be viewed as a threat to the self; *(3) acceptance* refers to the extent which young people incorporate their T1DM into their life and overall functioning. Young people are not overwhelmed by their condition; (4) enrichment refers to how diabetes results. It enables positivity in the individual's life, explicitly adding meaning to their identity.

Overall, Verschueren et al. (2020) and Commissariat et al. (2019) conclude by saying that it is necessary and essential to identify and support those young people who struggle to develop an identity, especially individuals who engage in active exploration but without a strong sense of commitment (Verschueren et al., 2020). Young people must ultimately be provided with the correct guidelines and tools to help them investigate/explore various options to engage and make firm commitments as they develop through different statuses (Verschueren et al., 2020).

Theme 2 Young People Living with T1DM can either Incorporate or Contain their Chronic Condition in their Identity Development

A study from Vanderhaegen et al. (2024) confirms theme 2. The authors explain that youth living with T1DM must find a way to integrate their chronic illness into their identity development, which is often referred to as illness identity (Vanderhaegen et al., 2024). Youth living with T1DM see their chronic condition as a threat to other aspects of themselves and tend to avoid thinking or talking about the impact it can have on their identity development and the meaning that it holds (Vanderhaegen et al., 2024). Commissariat et al. (2016) and Commissariat et al. (2019) suggest two ways young people can experience and manage T1DM through their identities: incorporating or containing the chronic condition. *Incorporation* refers explicitly to the young people who make their condition a part of who they are. They do not try to hide any aspects of their condition but merge it into their daily functioning and find ways to incorporate it into their identities (Commissariat et al., 2019). When young people can accept and incorporate T1DM into their daily lives and functioning, they can manage diabetes‐related burdens more successfully (Commissariat et al., 2016; Ingersgaard et al., 2024; Oris et al., 2016). Oris et al. (2016) argue the importance of incorporating T1DM into young people's identities and say that the incorporation of T1DM into one is deemed a crucial part of this period of the adolescent's life and development (Oris et al., 2016).

Previous studies have suggested that the extent to which young people incorporate their T1DM into their identities may affect their psychological and diabetes‐related functioning (Oris et al., 2016). Commissariat et al. (2019) further produce three significant components of incorporation: (1) self‐care when faced with stigma, (2) acceptance, and (3) sharing diabetes knowledge and experiences. Four studies (see Commissariat et al., 2016, 2019; Raymaekers et al., 2019; Verschueren et al., 2020) suggest that when T1DM is accepted and incorporated into their identities rather than contained, young people feel more assured about dealing with diabetes‐related issues, have higher levels of life satisfaction and apply more excellent self‐care. These four studies ultimately concluded that young people who incorporated their condition into their identity had much greater social competency and ability to interact with their environment.

Commissariat et al. (2016) further suggest three possible processes to help young people incorporate their T1DM into their identities. The first one is “becoming and being ill,” where they state that the individual must revise and redefine the sense of self as someone with a chronic condition (Commissariat et al., 2016). Secondly, *“*managing the condition” is where the person must incorporate or consolidate the new concepts of the condition and the sense of self by experiencing and understanding the changes within the body and mind (Commissariat et al., 2016). Moreover, “stigma and stigma control” is where the person must learn to discern and adapt to manage the actual stigma surrounding their chronic condition (Commissariat et al., 2016).

*Containment*, on the other hand, occurs when young people try to hide the T1DM diagnosis; they fear stigma from people and, lastly, try to maintain the identity that they had before their diagnosis (Commissariat et al., 2016). When T1DM is viewed as external to oneself, it can result in lower levels of well‐being and overall intensify the burden of living with T1DM (Commissariat et al., 2016). It is important to note that containment does not necessarily mean rejection; it simply means that adolescents have not decided whether or how to incorporate their T1DM into their sense of self (Commissariat et al., 2019; Raymaekers et al., 2019). The three studies by Commissariat et al. (2016), Commissariat et al. (2019) and Ingersgaard et al. (2024) highlight the continuous struggle young people face when they are trying to develop an identity that incorporates their T1DM so that they can become a “person with diabetes rather than a diabetic person.”—seeing themselves as an individual who will be able to live with their chronic condition rather than being defined by it.

One of the many burdens and challenges of living with T1DM is the stigma that young people deal with. For example, Commissariat et al. (2016) and Commissariat et al. (2019) state that when young people enter a new school/social situation, the individual will likely deny or contain their T1DM to form part of the peer group rather than being stigmatized for having T1DM. Health‐related stigma can negatively influence diabetes self‐management behavior, leading to poor treatment adherence and suboptimal glucose control (Jeong et al., 2018). Young people who have experienced health‐related stigma towards them have reported feelings of anger and psychological distress (Jeong et al., 2018). Considering the critical developmental stage of identity development in young people, they often lack the skills and capacity to cope with health‐related stigma (Jeong et al., 2018). However, when young people with T1DM can grasp and manage the actual and perceived stigma surrounding their chronic condition, it will be beneficial to their incorporation of diabetes into their identity (Commissariat et al., 2019).

It is important to note that young people with T1DM are more likely to be at risk for general and condition‐specific problems (Commissariat et al., 2016, 2019; Laursen et al., 2024; Raymaekers et al., 2019). Hence, young people living with T1DM are urged to incorporate their condition into their identity for healthy identity synthesis/development (Commissariat et al., 2019; Oris et al., 2016; Raymaekers et al., 2019). Commissariat et al. (2019) conclude that although the incorporation of T1DM is linked with higher levels of well‐being and social competency, it might not always be an indicator of fewer adverse reactions from friends or social groups; it ultimately comes down to the self‐perception of young people and the influence it has on their ability to incorporate T1DM into their identity.

Theme 3 External Factors Influence Identity Development in Young People Living with T1DM

Socio‐ecological theorists like Bronfenbrenner (1994) highlight that identity development occurs within dynamic systems by ongoing corresponding processes identified between the individual and the responses of their environment. Numerous factors contribute to young people's development and diabetes management as their environment interacts with them daily (Raymaekers et al., 2019). Two studies mentioned these factors, including personal and emotional feelings, family and peer dynamics, and other people's evaluations of them (Commissariat et al., 2019; Raymaekers et al., 2019). When young people living with T1DM go through adolescence and young adulthood, it coincides with a change in care responsibility from a family‐based approach to an individualized approach (Laursen et al., 2024). This transition typically tends to be a decrease in the quality of care.

Only two studies addressed young people and external factors influencing identity development. Raymaekers et al. (2019) report that *parents/caregivers* are critical in encouraging and developing young people's autonomy while helping them adapt and manage their diabetes. The study of Goethals et al. (2017) can be linked to that of Raymaekers et al. (2019) when they state that parents are directly connected and play a critical role in the caregiving of young people regarding diabetes‐related management. Suppose there is over‐involvement from the parents/caregiver. In that case, it is then likely for the autonomy‐seeking adolescent to perceive the demanding behavior as a hazard to their independence. The study further suggested that when the parents work *with* the adolescent rather than *controlling* their diabetes‐related issues, improved and positive changes can be seen in young people's identity development (Commissariat et al., 2016).

To illustrate, a study conducted by Overgaard et al. (2020) found that being a parent or caregiver of young people having T1DM can be very stressful because they will be in a constant state of vigilance trying to maintain the adolescent's diabetes and general well‐being. The study further suggests that diabetes management systematically shifts from the parent to the young people, becoming more of a partnership (Overgaard et al., 2020). According to Commissariat et al. (2016), it is essential to find the balance between the involvement of the parent without endangering young people's autonomy and independence, leaving room for collaboration on healthy diabetes management so that the adolescent can begin to develop a sense of self while living with this chronic condition.

Two of the included articles from Commissariat et al. (2016) and Raymaekers et al. (2019) state that young people living with T1DM quickly get upset when parents, caregivers or friends seem overly protective towards or worried about them. This may hinder the transfer of responsibility from the parents to the young people. This, in turn, can be linked to the confusion about the roles that each family member must fulfill because they need to learn or understand what is expected of them (Overgaard et al., 2020). The study's findings further propose that once T1DM is normalized, young people can freely express themselves and live as individuals with T1DM (Commissariat et al., 2016). Young people may sometimes experience people who focus more on their T1DM than on themselves as a person, which in turn can be viewed by the individual adolescent as an overall rejection of their sense of self (Raymaekers et al., 2019).

Although there are quite a few negative influences of T1DM, the study of Commissariat et al. (2016) found that young people were much happier and relieved when they disclosed their diagnosis to supportive friends and family, stating that young people indicated that social support from family and friends had a positive effect on how they lived with diabetes. Support from friends and family is essential, as identity development rests upon the continuous change and interaction between young people and their environment (Raymaekers et al., 2019). A study done by Overgaard et al. (2020) supports the positive side of having diabetes when they state that young people feel more appreciated by their families and that if they give the necessary attention to the chronic condition, it becomes manageable rather than disruptive to the family.

The study concludes that identity development can easily rely on constant social feedback from friends and family, depending on the daily choices that one may make (Overgaard et al., 2020). For instance, young people living with T1DM choosing to disclose their diagnosis to peers might not do it again if the social feedback they receive is negative. To put it differently, two of the studies found that when feedback does not align with one's identity, young people will be prone to change their behavior to gain new input or adapt their identity to find a solution to this inconsistent or uncomfortable state (Commissariat et al., 2019; Raymaekers et al., 2019).

## 5 IMPLICATIONS FOR YOUNG PEOPLE LIVING WITH T1DM

Ultimately, data from this critical review revealed that T1DM can be described as a fading or loss of the former self, making the demand to examine its effect on identity development significant (Lam et al., 2014). The individual living with T1DM experiences more difficulty and obstacles in forming and developing an identity (Oris et al., 2016; Vanderhaegen et al., 2024; Xing et al., 2015). The findings of this critical review confirm the study of Jaser and White (2011). They state that a T1DM diagnosis can generally lead to tremendous amounts of stress owing to the requirement of diabetes‐related treatments and management, the uncertainty of ways in which one can incorporate T1DM into one's identity or sense of self, and then, lastly, to shape a new reality of living life with a chronic condition such as T1DM.

The findings of this research study further suggest that although young people with T1DM must overcome numerous challenges, such challenges can still lead to positive outcomes. The studies of Commissariat et al. (2016), Overgaard et al. (2020) and Raymaekers et al. (2019) confirm this statement by reporting that young people feel more relieved and happier when they disclose their diagnosis to their peer groups because they feel more accepted when their friends understand their situation. Within their family context, young people felt that better physical and psychological management of the condition can lead to better balance within family relationships and that their parents and siblings appreciate them more (Raymaekers et al., 2019). Laursen et al. (2024) further imply that healthcare workers should pay attention to relational continuity and have a continuous caring relationship, which will help transition from pediatric to adult care.

It was also noticeable in the findings of this study as well as that of Jaser and White (2011), that young people must steer and negotiate between identity statuses all going through a developmental phase which is characterized by intense physical, emotional, cognitive and psychological change. Therefore, young people with T1DM will develop or form an identity unique to their chronic condition. As noted in the findings of the review and by Timler et al. (2019), young people who attempt to develop an identity despite living with a chronic condition have a stronger sense of belonging to peer groups, better attitude towards life and firmer belief systems (Timler et al., 2019). These, in turn, can be linked towards the notion concluded by various studies of young people gaining mastery over their lives and learning to balance demands (Jaser & White, 2011; Timler et al., 2019).

Although the study concludes and confirms that young people living with T1DM have numerous challenges that they must face, there are possibly a few interventions that can directly or indirectly help young people establish an identity despite having a chronic illness such as T1DM (Malik et al., 2020; Timler et al., 2019). Barry‐Menkhaus et al. (2020) propose multiple interventions to assist young people dealing with their chronic condition as well as the systems that are linked to them, for example, individual‐, friends‐, family‐, and school interventions for optimal physical and psychological functioning (Hilliard et al., 2017). Spencer et al. (2012) suggest that one such intervention can be technology‐based, helping youth with better knowledge and psychological well‐being and improving self‐care behaviors. Further research indicates that intervention combining experimental learning opportunities with technology could positively impact the youth's psychological well‐being (Spencer et al., 2012). Another intervention that has proven significant success is mind‐body interventions such as mindfulness programs and guided imagery (Weigensberg et al., 2018). However, it remains imperative to continue to develop interventions that will assist youth to maintain and control their T1DM throughout their lifetime (Weigensberg et al., 2018).

## 6 LIMITATIONS AND RECOMMENDATIONS OF THE STUDY

The reader needs to note that this critical review has certain limitations that must be considered. Different or more keywords could have been utilized to include a wider variety of data pools, and the search was limited to 2014 to 2024. Although developmental phases were explored within this review, specific outcomes regarding each developmental phase need to be identified for future studies. Throughout the research process, and precisely the data collection stage, it became evident that there is a lack of relevant and recent literature regarding identity development in young people living with T1DM. Some studies were outdated by more than 10 years and could not be included in the review. Although interventions are mentioned, there is still both a lack and need for interventions which specifically focus on identity development in young people living with T1DM. Therefore, this study recommends that future research focus on creating interventions for the latter.

## 7 CONCLUSION

In conclusion, this critical review sheds light on the identity development of young people living with T1DM. The review highlights that living with T1DM can significantly impact an individual's identity development, and the effects can be both positive and negative. The review also reveals that young people with T1DM develop their identity differently from those without chronic conditions. Furthermore, external factors such as caregivers and health professionals are crucial in shaping young people's identity development with T1DM.

In light of these findings, it is evident that more research studies are required to obtain data from young people living with T1DM as a primary source. This approach can help researchers better understand the experiences of young people with T1DM and their identity development. Lastly, the review highlights the need for healthcare professionals, caregivers, parents/guardians, and friends to appropriately support young people with T1DM in managing their condition, which can positively impact their identity development and overall psychological well‐being.

## CONFLICT OF INTEREST STATEMENT

The authors declare no conflict of interest.

## ETHICS STATEMENT

This study was approved by the Health Research Ethics Committee (HREC) of the North‐West University, South Africa, with approval number NWU‐00345‐20‐A1.

## Data Availability

All data is available in the manuscript as this is a critical review of previous literature. All other data relating to data and review processes can be requested from the corresponding author upon reasonable request.
